# An LSTM-Based Prediction Method for Lower Limb Intention Perception by Integrative Analysis of Kinect Visual Signal

**DOI:** 10.1155/2020/8024789

**Published:** 2020-07-23

**Authors:** Jie He, Zhexiao Guo, Ziwei Shao, Junhao Zhao, Guo Dan

**Affiliations:** ^1^School of Biomedical Engineering, Health Science Center, Shenzhen University, Shenzhen 518056, China; ^2^Zhejiang Provincial Hospital of Traditional Clinical Medical, Hangzhou 310006, China; ^3^Shenzhen Institute of Neuroscience, Shenzhen 518060, China

## Abstract

Recently, computer vision and deep learning technology has been applied in various gait rehabilitation researches. Considering the long short-term memory (LSTM) network has been proved an excellent performance in learn sequence feature representations, we proposed a lower limb joint trajectory prediction method based on LSTM for conducting active rehabilitation on a rehabilitation robotic system. Our approach based on synergy theory exploits that the follow-up lower limb joint trajectory, i.e. limb intention, could be generated by joint angles of the previous swing process of upper limb which were acquired from Kinect platform, an advanced computer vision platform for motion tracking. A customize Kinect-Treadmill data acquisition platform was built for this study. With this platform, data acquisition on ten healthy subjects is processed in four different walking speeds to acquire the joint angles calculated by Kinect visual signals of upper and lower limb swing. Then, the angles of hip and knee in one side which were presented as lower limb intentions are predicted by the fore angles of the elbow and shoulder on the opposite side via a trained LSTM model. The results indicate that the trained LSTM model has a better estimation of predicting the lower limb intentions, and the feasibility of Kinect visual signals has been validated as well.

## 1. Introduction

Stroke is a disease caused by acute rupture of blood vessels or vascular occlusion [1, 2]. About 15 million people suffer from it every year globally [3]. Hemiplegia is the major sequela of most stroke survivors which affects the quality of their daily life in the home, workplace, and community [4]. It presents with the weakness of one entire side of the body. Due to limb weaknesses leading to an inability to properly performing, hemiplegia patients could lose a number of motor functions especially the walking function [5, 6]. Walking abnormality makes performing everyday activities in the home, workplace, and community more difficult [7, 8].

Recovery of the walking ability for hemiplegia patients is crucial in order to perform daily activities [9, 10]. Key components of gait recovery are high-intensity, skill-oriented, and task-specific [11, 12]. Due to physically exhaustion of therapists to repeat hundreds of complex gait cycles in a training session [13], an amount of rehabilitation gait training robots have been developed to provide robotic assistance [14]. Robotic-assisted gait training refers to the rehabilitation therapists how to assist the patient in performing the gait cycle [15]. Considering the limb weaknesses leading to difficulty in supporting the body weight in training, current rehabilitation could support body weight to allow the lower limbs to maintain a pattern during gait training such as Lokomat [16]. These gait robot trainers passively move the patients on a treadmill. However, the control systems of most commercial robotic systems are passive in nature because the training subject is not considered in the system. By increasing active participation [17], the dependence of patients on robot assistance can be reduced by improving the effectiveness of rehabilitation training. Thus, we should make the robots include the ability that collects quantitative gait data to generate sensory stimulation synchronized to gait patterns.

To develop a noncontact signal prediction for an active rehabilitation robotic system, the synergy that is initiated by weight-bearing over the involved limb and supporting the human body was taken into consideration [18, 19]. Twitchell et al. proved that abnormal synergy is a motor impairment in patients with stroke [20]. The main factor that limited the motor rehabilitation of patients with stroke is abnormal synergy [21]. Studies have shown that interlimb and intralimb coordination of lower limbs in patients after stroke is diverse from that in normal subjects [22]. In 2018, Zebin et al. proposed a prediction method via inertial sensors and LSTM methods to predict the angle trajectory of the impaired lower limb [23]. Simultaneously, the security and privacy of medical data are also crucial. Sandeep et al. developed a biometric-based security framework for wearable health monitoring systems to extract ECG signal, and it proved that time-domain based biometric features plays an important role in security [24]. Wu et al. proposed an adaptive computing-based random binary sequences generation method to provide a balance between processing time and security in wireless body sensor networks [25]. Cai et al. quantified the concurrent accuracy and the test-retest reliability of a Kinect V2-based upper limb functional assessment system [26]. Liao et al. proposed a motion intention recognition system based on the Kinect V2 sensor. It can successfully provide an adequate assistance with a lesser time delay compared with the system without Kalman filter [27].

Recently, the time series prediction model has been effectively applied to several studies [28]. Long short-term memory (LSTM) networks widely used to have done a good job on this issue in fields including gait recognition owing to the ability of processing and predicting the time series with very long intervals [29, 30]. It works effectively to extract the gait feature [11].

**Figure 1:**
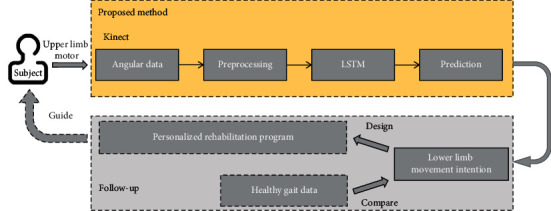
The framework of our method and follow-up studies.

As shown in Figure 1, in this paper, a lower limb joint trajectory generation framework was proposed to drive the lower limb robot using the trajectory of healthy upper limbs. This study aimed to utilize upper limb Kinect information during walking to estimate sagittal plane hip and knee kinematics trajectories. The trajectories will be used for driving a rehabilitation robotic system in follow-up studies.

## 2. Methods

### 2.1. Experimental Setup and Data Acquisition

**Figure 2:**
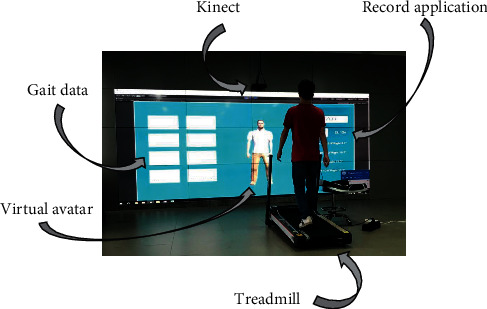
Treadmill and Kinect layout (the treadmill was angled at 45 with respect to the Kinect sensor, with the front of the treadmill positioned 140 cm to the right and at a distance of 150 cm in front of the sensor; the base of the Kinect sensor rested 100 cm above the floor).

To obtain human gait data, we have built and evaluate our model that used a “virtual skeleton” produced by the Kinect sensor and software. Kinect 2.0 provides a high-quality skeletal model to one user in front of the Kinect sensor, and Kinect SDK offers the tracking and detection of 25 different skeletal points, which could apply this skeletal data for feature extraction; the experimental setup is as shown in Figure 2.

Gait data were concurrently recorded by a Kinect sensor that provides approximately 30 skeleton frames per second [31]. Each participant wore a fitting and light color suit on the treadmill. In a 10 participants' database, they are generally divided into four walking velocities: 3.0, 3.5, 4.0, and 4.5 km/h.

The joint angle of the shoulder, ankle, hip, knee, arm of the right side, left knee, and hip in the sagittal plane were calculated based on the quaternion. For each joint of the Kinect virtual model, the *x*, *y*, and *z* coordinates are recorded. This study converts the joints into a vector for angle calculation. For each joint, the current position of the angle between a joint and a sagittal vector was recorded. Finally, we generate the following features: the angle in each of the frames, the difference in angle between consecutive frames, and these angular displacements providing basic gait characteristics.

### 2.2. Gait Joint Angle Design

**Figure 3:**
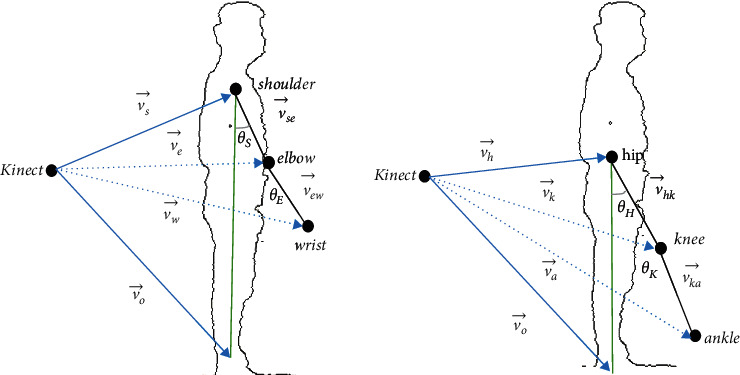
Determination of *θ*.

The Kinect skeletal joints 3-D coordinated data reading is less susceptible to noise compared with their distance to the acquisition [32, 33]. Thus, for each limb, a shoulder joint angle was determined by considering the location of the shoulder and elbow in the Cartesian coordinate. The shoulder, elbow, hip, and knee position in Cartesian space are defined with four-vectors, the Kinect being at the origin of the 3-D space. The vector definition is formulated in equations (1)–(5). The angle of joints definition is formulated in equations (6)–(9):

(1) $$\begin{array}{c} {\overset{⟶}{v}}_{se}={\overset{⟶}{v}}_{s}-{\overset{⟶}{v}}_{e}, \end{array}$$

(2) $$\begin{array}{c} {\overset{⟶}{v}}_{ew}={\overset{⟶}{v}}_{e}-{\overset{⟶}{v}}_{w}, \end{array}$$

(3) $$\begin{array}{c} {\overset{⟶}{v}}_{hk}={\overset{⟶}{v}}_{h}-{\overset{⟶}{v}}_{k}, \end{array}$$

(4) $$\begin{array}{c} {\overset{⟶}{v}}_{ka}={\overset{⟶}{v}}_{k}-{\overset{⟶}{v}}_{a}, \end{array}$$

(5) $$\begin{array}{c} {\overset{⟶}{v}}_{\text{sag}}={\overset{⟶}{v}}_{s}-{\overset{⟶}{v}}_{o}. \end{array}$$

(6) $$\begin{array}{c} \theta_{S}=\;\cos^{-1}\left({\overset{⟶}{v}}_{se}\cdot \overset{⟶}{v_{\text{sag}}}\right), \end{array}$$

(7) $$\begin{array}{c} \theta_{E}=\;\cos^{-1}\left({\overset{⟶}{v}}_{ew}\cdot {\overset{⟶}{v}}_{\text{sag}}\right), \end{array}$$

(8) $$\begin{array}{c} \theta_{H}=\;\cos^{-1}\left({\overset{⟶}{v}}_{hk}\cdot {\overset{⟶}{v}}_{\text{sag}}\right), \end{array}$$

(9) $$\begin{array}{c} \theta_{K}=\;\cos^{-1}\left({\overset{⟶}{v}}_{ka}\cdot {\overset{⟶}{v}}_{\text{sag}}\right), \end{array}$$

where ${\overset{⟶}{v}}_{se}$, ${\overset{⟶}{v}}_{ew}$, ${\overset{⟶}{v}}_{hk}$, and ${\overset{⟶}{v}}_{ka}$ are the 3-D vectors connecting the participants' shoulder to the elbow, elbow to the wrist, hip to the knee, and knee to the ankle, respectively, that is also depicted in Figure 3.

### 2.3. Long-Short Term Memory Network for Angle Prediction

In our proposed approach, trajectory generation is to apply the interlimb synergy extracted from healthy participants by LSTM to generate a trajectory-based on gait data [34, 35].

**Figure 4:**
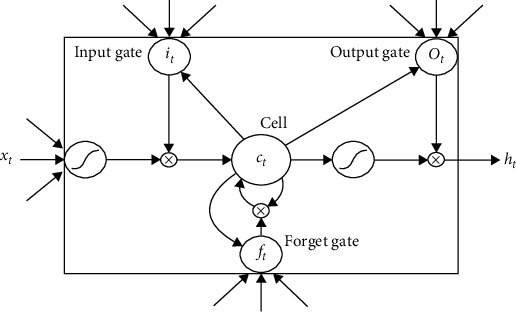
The structure of an LSTM neuron [36].

To solve the difficulties in training the RNN model caused by the “vanishing gradient” effect, the long-short term memory (LSTM) architecture has been proposed.  Figure 4 illustrates a typical LSTM neuron. It contains one self-connected memory cell *c*_*t*_ and three multiplicative units, i.e., the input gate *i*_*t*_, the forget gate *f*_*t*_, and the output gate *o*_*t*_.

The memory cell has a self-connected recurrent edge of weight 1, ensuring that the gradient can pass across many time steps without vanishing or exploding [29]. The input gate and forget gate govern the information flow into and out of the cell [37]. The output gate controls how much information from the cell is passed to the output *h*_*t*_. The activations of the memory cell and three gates are given as follows:

(10) $$\begin{array}{c} i_{t}=\sigma \left(W_{xi}x_{t}+W_{hi}h_{t-1}+W_{ci}c_{t-1}+b_{i}\right),\\ f_{t}=\sigma \left(W_{xf}x_{t}+W_{hf}h_{t-1}+W_{cf}c_{t-1}+b_{f}\right),\\ c_{t}=f_{t}c_{t-1}+i_{t}\;\tanh\left(W_{xc}x_{t}+W_{hc}h_{t-1}+b_{c}\right),\\ o_{t}=\sigma \left(W_{xo}x_{t}+W_{ho}h_{t-1}+W_{co}c_{t-1}+b_{o}\right),\\ h_{t}=o_{t}\tanh\left(c_{t}\right). \end{array}$$

where *σ*(*x*) is the logistic sigmoid function and defined as *σ*(*x*)=1/(1+*e*^−*x*^), *w*_*αβ*_ are the weight matrices connecting *α* and *β*, and *b*_*β*_ denotes the corresponding bias vectors.

## 3. Experiment

### 3.1. Experiment Implementation

Since stroke patients show a lower extremity weakness of walking [38, 39], we target in studying the spatial correlations of gait features by using neural networks. To get enough training gait data, 10 healthy participants (aged 23.3 ± 1.4years, height 169.1 ± 6.9cm, and weight 55.5 ± 6.5kg) were recruited from our laboratory. They were free of any physical condition or limitation which prevented them from walking on the treadmill. They were required to walk for 150s per velocity.

For maintaining a stable recognition of the human body [40], the Kinect was placed at a height of 1 meter above the ground and the treadmill was set within 2.6 to 4 meters from the Kinect sensor.

**Figure 5:**
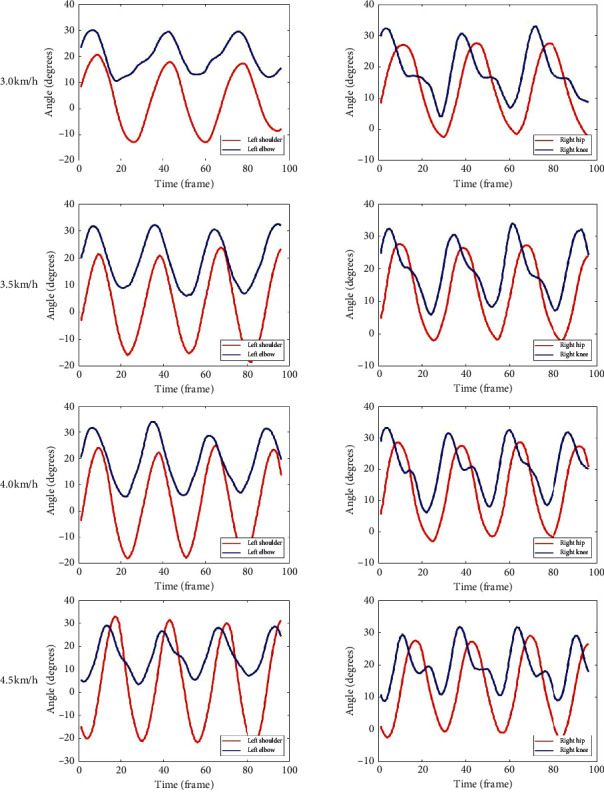
Left upper limb joint angle and right lower limb joint angle during walking at four velocities.

During the experiment, there was a total of 10 (male/female:6/4) healthy participants enrolled. We prepared 40 gait feature data of upper and lower limbs from 10 subjects, while their skeletal data were captured by Kinect 2.0.  Figure 5 illustrates a participant walking session and joints behavior during a gait cycle in different velocities.

Our experiments were implemented on the Tensorflow framework [36], a popular deep learning framework. The base learning rate was set to 0.0005, and the LSTM step size was set to 10 frames. The maximum number of iterations was set to 1000.

### 3.2. Results

**Figure 6:**
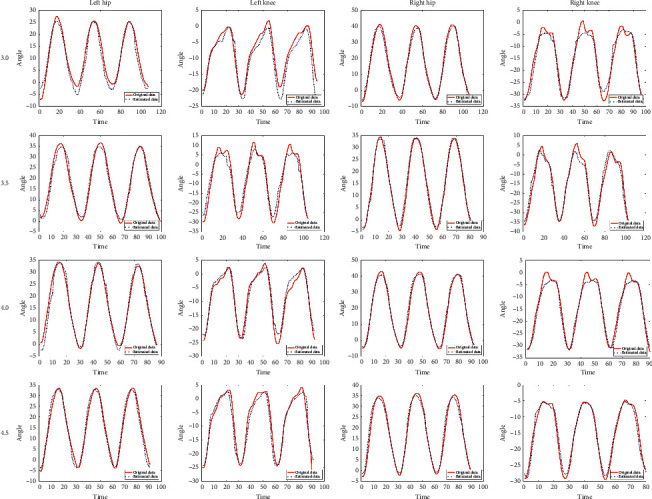
Estimated left hip and knee trajectories vs. original hip and knee trajectories through using the right shoulder and elbow in different velocities (3.0, 3.5, 4.0, and 4.5 km/h).

**Figure 7:**
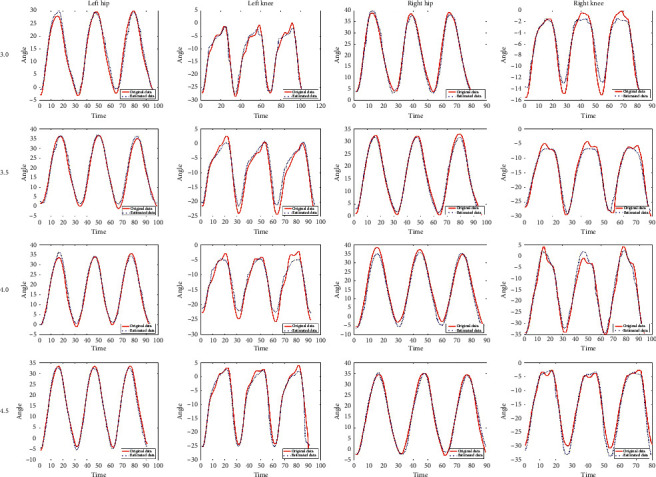
Estimated one side's hip and knee trajectories vs. original hip and knee trajectories through using the other side's shoulder, elbow, hip, and knee in different velocities (3.0, 3.5, 4.0, and 4.5 km/h).

This study estimated one side's gait data by using the other side's data based on the synergy.  Figure 6 shows the estimated result of left hip joint and knee joint trajectories through using the right shoulder and elbow by LSTM. To validate the feasibility of LSTM synergy, we used right side upper limb joints and lower limb joints to predict left side lower limb and are shown in Figure 7; it shows the estimated result of one side's hip and knee trajectories through using the other side's shoulder, elbow, hip, and knee by LSTM. As can be seen from the figure, the error between the estimated trajectory and the original trajectory of the left hip and knee is low.

**Figure 8:**
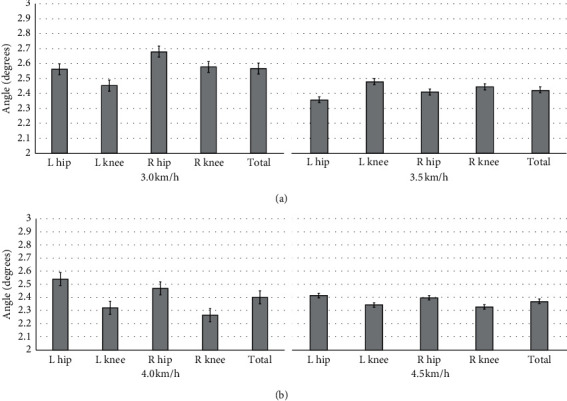
RMSE of LSTM estimation on hip and knee extension and flexion using the right shoulder and elbow in different velocities for Kinect.

**Figure 9:**
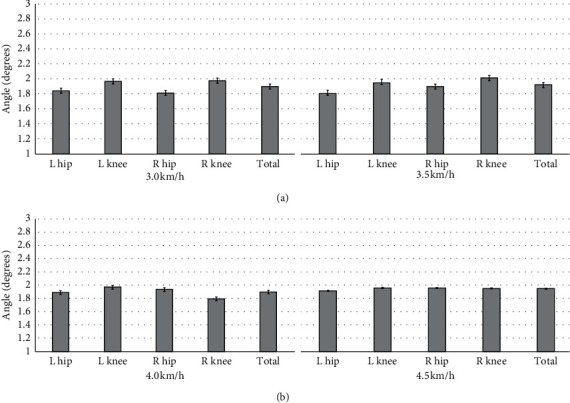
RMSE of LSTM estimation on hip and knee extension and flexion using the shoulder, elbow, hip, and knee in different velocities for Kinect.

Results show that LSTM is a good approach for person identification based on gait recognition with Kinect. We also tested the quality of the prediction of the angular velocity, and we applied the root-mean-squared error (RMSE) to evaluate the model after each run. Here, we compared RMSE between the estimated angle and original angle in four different velocities on prediction based on LSTM. The result is shown in Figures 8 and 9. Especially, RMSE was poor for hip and knee joint angles at 3.0 km/h than the other three velocities by using the joints of the upper limb (Table 1); however, it was relatively good by using the joints of the upper limb and lower limb (Table 2).

## 4. Discussion

To estimate the hip and knee trajectory by using the upper limb joints trajectories, we applied the Kinect 2.0 to track the upper limb and lower limb sagittal plane movement in the walking period. The human body is in a continuous dynamic state during walking. In this study, the LSTM model was developed, and its performances were compared using RMSE. Because there is no need to go through a process of selecting features and having better stability, we chose it to estimate our trajectory. The LSTM model in this study showed improved results, and RMSE has been introduced above. It can see that LSTM has a better estimation on predicting the gait trajectory, which included human interlimb synergy. This model showed excellency in modeling that with the change over time such as walking to predict the data of current time from information in the previous step.

As the pace velocity increases, we can see that the accuracy of the prediction is getting higher. In the case of 3.0 km/h velocity, the gait prediction trajectory is relatively poor; however, in the case of 4.5 km/h velocity, LSTM presents the effect of prediction is quite amazing. This result indicates that when humans walk at 4.5 km/h velocity, the upper and lower limbs on the two sides are highly correlated.

In various velocities, the trajectory prediction effect of the knee joint is generally higher than that of the hip joint, except for the velocity in 3.5 km/h.

When using the joints of the upper limb and lower limb to estimate the hip and knee trajectory, we can get an obvious better estimation accuracy. From Table 2, we can see that the RMSE is basically maintained within 2, which is better than merely used the upper limb to predict hip and knee trajectory. Otherwise, in this case, the accuracy of the estimated hip trajectory is better than estimated knee trajectory, respectively; compared with the right hip, the left hip trajectory is great. In Figure 8, the trajectory only based on the upper limb trajectory still has a good estimation performance. It was concluded that LSTM has good exploitation in gait features.

This study has a limitation of not applying data of patients with stroke to the learning model for lower limb trajectory prediction. However, the study is to suggest the possibility of estimating the lower limb trajectory by using the upper limb trajectory and an artificial neural network model. In the next research, we can apply various data for the training model.

## 5. Conclusion

In this paper, an artificial neural network model was developed to estimate the lower limb joints trajectory of a complete gait cycle by using the joints of the opposite side. Accuracies of using the upper limb joints and the upper and lower limb joints to estimate another side lower limb joints were compared. As a result, the model showed RMSE values within 3.0. These trials demonstrate that this model can be used safely as a gait training intervention for those stroke patients. It suggests that the exoskeletal gait rehabilitation robot can apply this model to help patients try to walk like normal people.

## Figures and Tables

**Table 1:** Mean and standard deviation at four velocities for Kinect by using the joints of the upper limb.

	3.0 km/h	3.5 km/h	4.0 km/h	4.5 km/h
L hip	2.56 ± 0.89	2.36 ± 0.69	2.54 ± 0.91	2.41 ± 0.58
L knee	2.45 ± 0.78	2.48 ± 0.70	2.32 ± 0.51	2.34 ± 0.64
R hip	2.68 ± 0.76	2.41 ± 0.65	2.47 ± 0.33	2.39 ± 0.57
R knee	2.58 ± 0.81	2.45 ± 0.54	2.26 ± 0.57	2.33 ± 0.69
Total	2.57 ± 0.81	2.43 ± 0.65	2.39 ± 0.58	2.37 ± 0.62

**Table 2:** Mean and standard deviation at four velocities for Kinect by using the joints of the upper limb and lower limb.

	3.0 km/h	3.5 km/h	4.0 km/h	4.5 km/h
L hip	1.84 ± 0.37	1.82 ± 0.39	1.89 ± 0.59	1.92 ± 0.36
L knee	1.97 ± 0.35	1.96 ± 0.44	1.97 ± 0.38	1.96 ± 0.43
R hip	1.81 ± 0.26	1.90 ± 0.43	1.94 ± 0.41	1.96 ± 0.29
R knee	1.98 ± 0.55	2.02 ± 0.33	1.79 ± 0.25	1.95 ± 0.47
Total	1.90 ± 0.38	1.93 ± 0.39	1.89 ± 0.41	1.95 ± 0.38

## Data Availability

The raw data required to reproduce these findings cannot be shared at this time as the data also forms part of an ongoing study.
